# Estimated benefits of providing on-demand pre-exposure prophylaxis options for cisgender women in South Africa: A modeling study

**DOI:** 10.1371/journal.pone.0357094

**Published:** 2026-08-27

**Authors:** Sarah E. Stansfield, Mia Moore, Marie-Claude Boily, James P. Hughes, Deborah Donnell, Linda-Gail Bekker, Dobromir T. Dimitrov

**Affiliations:** 1 Vaccine & Infectious Disease Division, Fred Hutchinson Cancer Center, Seattle, Washington, United States of America; 2 School of Public Health, Imperial College London, London, United Kingdom; 3 Department of Biostatistics, University of Washington, Seattle, Washington, United States of America; 4 Department of Global Health, University of Washington, Seattle, Washington, United States of America; 5 The Desmond Tutu HIV Centre, University of Cape Town, Cape Town, South Africa; 6 Department of Applied Mathematics, University of Washington, Seattle, Washington, United States of America; Robert Koch Institut, GERMANY

## Abstract

**Introduction:**

On-demand oral tenofovir disoproxil fumarate–emtricitabine (TDF/FTC) pre-exposure prophylaxis (PrEP) has been shown to be effective at preventing HIV acquisition among cisgender men and transgender women but is not recommended for cisgender women. On-demand PrEP may improve PrEP uptake, effective use, and persistence in cisgender women. We utilized published oral PrEP adherence-efficacy curves and data from HPTN 067 study to estimate effectiveness of different non-daily PrEP options when used by cisgender women and investigate which sub-groups may benefit most from on-demand PrEP.

**Methods:**

We created a synthetic cohort of PrEP users with data on sex act frequency and pill taking from the HPTN 067 Cape Town site. We simulated PrEP use with three PrEP regimens tested in HPTN 067: daily, time-driven (2 pills/week+1 pill after sex), and event-driven (1 pill before+1 pill after sex) PrEP, and hypothesized 2-1-1 PrEP (2 pills before+1 pill each of the two days after sex) for six months each. We estimated PrEP effectiveness based on the number of pills taken around sex acts. Adherence to 2-1-1 PrEP, which was not tested in the HPTN 067, was informed by observed adherence to event-driven PrEP. Assignment to 2-1-1 PrEP based on daily PrEP adherence and sex act frequency was also analyzed.

**Results:**

We estimated median effectiveness of 86% for daily, 47% for time-driven, 42% for event-driven, and 57% for 2-1-1 PrEP. PrEP users with low adherence to daily PrEP (less than 2.8 pills per week) benefited most from on-demand PrEP. In this subgroup, comprising 8% of the cohort, PrEP effectiveness increased from 41% to 44% when switching from daily to 2-1-1 PrEP while pill taking decreased from 2.2 to 1.4 pills per week; population-level effectiveness was unchanged when this subgroup switched to on-demand PrEP. We found no advantage in assigning 2-1-1 PrEP by sex act frequency.

**Conclusions:**

Our model suggests that on-demand PrEP could benefit women with low daily PrEP adherence by decreasing the number of days when pills need to be taken and modestly increasing effectiveness compared to a daily regimen. This would be a valuable and easy-to-implement additional option in places where daily oral PrEP is already available.

## 1. Introduction

Multiple types of pre-exposure prophylaxis (PrEP) have shown efficacy in preventing HIV acquisition among cisgender women in randomized-controlled trials including daily oral pills (oral tenofovir disoproxil fumarate/emtricitabine, TDF/FTC), topical (vaginal rings), and long-acting injectable (cabotegravir, CAB-LA, and lenacapavir, LEN) regimens [1–5]. These options should empower more women to find HIV prevention that suits their needs and preferences. Within the oral PrEP options, both daily PrEP and 2-1-1 PrEP (2 pills before and 1 pill each of the following two days after sex) have demonstrated efficacy for cisgender men who have sex with men (MSM) and transgender women (TGW) [6], but only daily PrEP is currently recommended for cisgender women [7].

While daily PrEP is very effective for women when adherent, safety factors such as intimate partner violence and time factors such as childcare and domestic labor act as barriers to high adherence [8,9]. Stigma, misinformation, and delayed clinic uptake of PrEP also create serious challenges to successful daily PrEP use [10–12]. In addition to adherence challenges, cisgender women also appear to have less dosing forgiveness than MSM/TGW [13], potentially due to differences in drug distribution to rectal versus vaginal tissue [14], though this has been recently called into question [15]. For MSM, PrEP coverage levels have been associated with efficacy based on an adherence-efficacy curve that had been previously established with data from the iPrEX study [16]. Until recently there was no equivalent dose-efficacy curve for cisgender women. Quantifiable levels of plasma tenofovir, as a marker of short-term adherence, had been established as a correlate of protection, confirming that daily oral PrEP should be effective for women if taken as prescribed [17]. In the last two years, several independent studies utilized the increasing amount of data from controlled usage of oral PrEP to propose adherence-efficacy curves for women [18–21]. These analyses generally supported the prevailing opinion that oral PrEP is less forgiving for doses missed by women [13] and provoked a debate on the feasibility of offering different on-demand PrEP regimens to cisgender women [22]. This is critical, as one recent systematic review of Sub-Saharan African women’s PrEP preferences found that adherence to daily oral PrEP was a barrier to many women and that some women were very interested in on-demand PrEP products, especially women who could accurately predict sex acts [23]. Adolescents in Uganda reported preferences for on-demand oral PrEP if they had less frequent sex acts [24] and young people in South Africa, Uganda, and Zimbabwe preferred on-demand oral PrEP for reasons such as stigma, pill fatigue, and adherence challenges [25].

The HPTN 067 study tested additional non-daily options among cisgender women in Cape Town, South Africa (enrolled from 2011–2012) and among MSM in Harlem, New York, USA, and Bangkok, Thailand (enrolled from 2012–2014) [26,27]. Daily PrEP was compared to time-driven PrEP (one pill twice per week with a post-sex dose 0–2 hours after sex) and event-driven PrEP (1 pill 24–48 hours before and 1 pill 0–2 hours after sex). All PrEP regimens used TDF/FTC. Detailed PrEP adherence and sexual behavioral data was collected with electronic monitoring of pill bottles opening and weekly interviews on pill taking and sexual activity and with pharmacokinetic monitoring [26,27]. Women in the HPTN 067 Cape Town site had unusually high adherence to daily PrEP compared to other trials in cisgender women [28], with 75% of sex acts reported covered by PrEP and drug detected in 68% of samples where sex had been reported in the previous week [26]. Adherence to the non-daily PrEP regimens was lower than to daily PrEP, with 56% and 52% of sex acts reported covered and drug detected in 58% and 41% of samples where sex had been reported in the previous week in the time- and event-driven arms, respectively.

In this study, we use mathematical modeling to estimate sexual activity and pill taking with daily, time-driven, event-driven, and 2-1-1 PrEP and evaluated the overall PrEP effectiveness in a simulated cohort resembling HPTN 067 Cape Town participants. A key feature of our modeling framework is the ability to predict adherence to multiple oral PrEP regimens based on actual trial data. We studied associations between the probability of taking regularly scheduled pills and pills around expected sex acts, sexual behavior, and ability to predict sex acts to estimate how individuals would respond to different assigned PrEP regimens. We explored a “person-centered care” scenario in which either daily or 2-1-1 PrEP was offered to individuals based on adherence to daily PrEP, aiming to identify prevention strategies with the highest effectiveness. This work is an extension of a previously published analysis evaluating a similar person-centered approach when daily and on-demand PrEP options were provided to MSM in the US and Thailand [29]. The ideal PrEP option for an individual may depend on factors including sexual behavior patterns, likelihood of high adherence, and personal preference. A quantitative framework, relating these factors and personal effectiveness can potentially aid providers in suggesting person-centered optimal options as efficacy alone is often not the deciding factor impacting individual choice of regimen [30].

## 2. Methods

We used a stochastic individual-based model to simulate sexual activity, TDF/FTC PrEP use, and PrEP effectiveness in a synthetic cohort of 10,000 female PrEP users assigned to one of four PrEP regimens (daily, time-driven, event-driven, or 2-1-1 PrEP, Table 1). Sex acts and pills taken, including pills taken close to sex, are simulated each day for each participant based on a HPTN 067 participant’s sex act and pill taking data (see Fig S1 in S1 File for the distribution of participant sex acts and Fig S2 in S1 File for the distribution of PrEP adherence levels). Mean PrEP adherence was 58% and mean sex acts per week was 1.4. PrEP effectiveness is then estimated based on the alignment of pill-taking and sexual activity.

**Table 1:** PrEP regimen descriptions.

PrEP Regimen	Pill taking	Adherence type	Data source
**Daily**	1 pill/day	Fixed-pill adherence	HPTN 067
**Time-driven**	2 pills/week +1 pill after sex	Fixed-pill adherence & on-demand adherence	HPTN 067
**Event-driven**	1 pill before +1 pill after sex	On-demand adherence	HPTN 067
**2-1-1**	2 pills before +1 pill each of the two days after sex	On-demand adherence	Hypothetical data: adapted from the event-driven regimen

### Simulating PrEP use

To predict PrEP use in each regimen, we used a generalized-linear mixed model framework originally developed for the MSM populations enrolled in the HPTN 067 trial [29] and recalibrated here for cisgender women, using data from Cape Town study site. Data on pill taking came from WisePill pill cases that transmitted times of opening. Weekly interviews confirmed pill taking times and determined timing of sex acts. Each woman in the cohort has a set of pill-taking probabilities within discrete time intervals defined by sexual activity and recent pill-taking (Table 2 & S1.1 in S1 File). The 2-1-1 PrEP regimen is a special case, as HPTN 067 did not test this regimen. Therefore, we adapted the event-driven regimen to inform 2-1-1 PrEP by applying the pre- and post-sex pill taking probabilities estimated there to simulate the pre-sex and first post-sex pill taking probabilities respectively for 2-1-1 PrEP. We assumed that if the first post-sex pill was taken, the second modeled pill was taken on the following day with a 90% probability [31].

**Table 2:** Pill taking probability by interval type.

	Interval Type	Pill Taking Probability	
		Median	IQR
A. Daily PrEP^1^	Pill taken day before	84%	71 - 92%
	Pill not taken day before	63%	49 - 75%
B. 2-1-1 PrEP^2^	No sex act day before, day of, day after	6%	3 - 11%
	2-24 hours prior to sex act	23%	16 - 31%
	0-2 hours prior to sex act, pill taken in 2–24 hours prior to sex interval	18%	13 - 25%
	0-2 hours prior to sex act, pill not taken in 2–24 hours prior to sex interval	19%	5 - 54%
	Day 1 after sex act, pre-sex act pill taken	28%	15 - 48%
	Day 1 after sex act, pre-sex act pill not taken	8%	3 - 18%
	Day 2 after sex act, day 1 after sex act pill taken^3^	90%	NA
	Day 2 after sex act, day 1 after sex act pill not taken	8%	3 - 18%

A: Daily PrEP pill taking probabilities, estimated with HPTN 067 daily arm data. B: 2-1-1 PrEP pill taking probabilities estimated with HPTN 067 event-driven arm data, except for the Day 2 after sex interval, which had probability assigned from 2-1-1 PrEP adherence data.^11^ Pill taking intervals are illustrated in Fig S4 in S1 File. Table including time-driven and event-driven arms in Supplement, Table S1 in S1 File.

^1^From HPTN 067 daily arm

^2^From HPTN 067 event-driven arm

^3^From literature [31]

The individual-level PrEP effectiveness was calculated on a per-sex act basis as the percent reduction in HIV risk, accounting for the predicted number of pills taken around each sex act. We assumed that the risk of HIV acquisition per sex act was reduced by 59.3%, 83.8%, or 95.9%, respectively, if at least two, four, or seven cumulative pills were taken within a week around that act (extending from day −4 to day + 2 around the sex act) [19]. The effectiveness for each individual was the median level of protection across all sex acts and similarly the population-level effectiveness was the median level of protection across all sex acts in the simulated cohort. Full details of the methods can be found in the Supplement and have been previously described [29]. Ethical approval was not required for this work.

### Simulating PrEP effectiveness with single regimens

We first simulated four synthetic cohorts, one for each of the modeled PrEP regimens. Sexual activity and pill taking behaviors were estimated from participants on each HPTN 067 regimen (daily, time-driven, and event-driven) and used to create distributions of these behaviors for individuals assigned to these regimens and to 2-1-1 PrEP. All individuals in simulated cohorts were assigned to the same PrEP regimen for six months to estimate effectiveness of that regimen.

### Simulating PrEP effectiveness with personalized regimens

**Fig 1:**
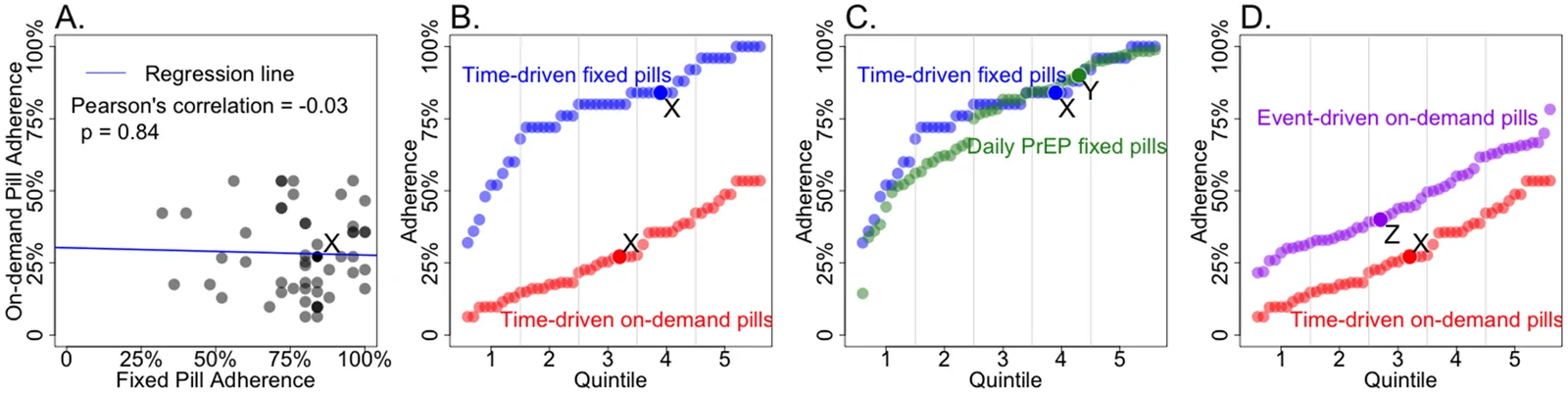
Bridging between PrEP regimens. Plots show data from the HPTN 067 trial. A: Fixed and on-demand pill adherence in the time-driven arm of HPTN 067. Each point shows one participant’s adherence values. When a model individual is created, a participant from the time-driven arm of HPTN 067 is sampled (Participant X). B: Fixed and on-demand pill adherence in the time-driven arm sorted by increasing adherence. Participant X has fixed and on-demand PrEP adherence values in quintiles 4 and 3 respectively. C: Fixed pill adherence in the time-driven arm and fixed pill adherence in the daily PrEP arm, sorted by increasing adherence. For fixed adherence, a participant in the daily PrEP arm with adherence also in quintile 4 is sampled (Participant Y) and the modeled individual has daily PrEP adherence equal to that participant. D: On-demand pill adherence in the time-driven arm and on-demand pill adherence in the event-driven arm, sorted by increasing adherence. For on-demand adherence, a participant in the event-driven PrEP arm with post-sex pill adherence in quintile 3 is sampled (Participant Z) and the modeled individual has on-demand PrEP adherence equal to that participant.

We also simulated a cross-over trial of daily and 2-1-1 PrEP. To connect these two PrEP regimens, we analyzed HPTN 067 data on two distinct pill-taking behaviors: 1) fixed-pill adherence, i.e., pills taken at regular intervals (daily or weekly) versus 2) on-demand adherence, i.e., pills taken before and after sex acts. Data from the time-driven arm of HPTN 067 (Fig 1A), which required participants take both weekly pills (fixed) and pills after sex (on-demand), was used to infer individuals’ behavior as they switch between regimens. We assumed that when an individual created after a time-driven HPTN 067 participant was reassigned to a new regimen (daily or 2-1-1 PrEP), she will retain her relative adherence ranking — that is, remain in the same quintile, with respect to on-demand and fixed pill adherence. To construct this cohort, we used model individuals whose PrEP parameters correspond to the posterior modes when fitting the model to specific HPTN 067 participants. We used the following algorithm (Fig 1, expanded in Fig S3 in S1 File):

1. We sampled a modeled participant from the time-driven arm of HPTN 067 (Participant X in Fig 1). Participant X had both fixed and on-demand PrEP adherence values (in quintiles A and B, respectively).
2. We sampled a modeled participant from the daily PrEP arm (Y) with adherence also in quintile A.
3. We sampled a modeled participant in the event-driven PrEP arm (Z) with adherence in quintile B.
4. Our new modeled individual behaves like Y while taking daily PrEP and Z while taking on-demand PrEP.

This synthetic cohort was modeled with all individuals on daily PrEP for six months followed by individual-specific personalized assignments for six months. We evaluated an adherence-based PrEP assignment in which only individuals with low adherence to daily PrEP (fewer than 40% of prescribed pills taken) were assigned 2-1-1 PrEP while the rest were assigned to daily PrEP.

### Sensitivity analyses

We tested the robustness of the adherence-efficacy curve by (for the upper bound of PrEP efficacy) using efficacy values derived from Marrazzo et al. (61.6%, 89.8%, and 100% reduction in HIV incidence with two, four, and seven pills/week) [20] and assuming 100% efficacy for acts around which 2-1-1 PrEP was taken correctly and (for the lower bound of PrEP efficacy) using the lower bound of the 95% creditable interval (29.9%, 51.7%, and 72.6% reduction in HIV incidence with two, four, and seven pills/week) [19]. We also removed from the analysis participants whose pharmacokinetic data did not match their pill-taking reports. A sensitivity analysis was conducted for the adherence-based PrEP assignment in which 2-1-1 PrEP was assigned to individuals with daily PrEP adherence equivalent to taking less than four pills per week (below 57% pills taken). Finally, we evaluated the effectiveness of an “optimal” PrEP assignment in which we assign either daily or 2-1-1 PrEP for each individual based on calculated individual effectiveness and estimated difference in pill burden (full description in Supplement) to be able to compare feasible scenarios with the most ideal scenario of PrEP assignment.

Linda-Gail Bekker had access to information that could identify individual participants during or after data collection but was not involved in model creation. No other authors had access to information that could identify individual participants.

## Results

### Pill taking behavior by cisgender women in HPTN 067

Cisgender women participants in the daily arm in Cape Town had a higher probability of taking a pill if one was taken the previous day than if it was missed (median probability: 84% vs 63%, Table 2A). This indicates somewhat different pill taking patterns compared to the Harlem study site where we estimated a larger difference between these probabilities (79% vs 46%), resulting in series of days in which all pills were taken or missed [29]. Pill taking behavior was estimated with HPTN 067 daily arm data.

Slightly more participants in the event-driven arm (used here to inform the pill-taking probabilities for 2-1-1 PrEP) took pills in the prescribed 2–24 hour window before sex than in the 0–2 hours before sex without a previous pill (median probability: 23% vs 19%, Table 2B). Eighteen percent of the participants who took a pill 2–24 hours before sex also took an additional pill 0–2 hours before sex. Participants were more likely to take a post-sex act pill in the prescribed day after sex if they had taken a pre-sex pill than if they had not (median probability: 28% vs 8%). Pill taking behavior was estimated with HPTN 067 event-driven arm data. Pill taking intervals are illustrated in Fig S4 in S1 File. Pill taking before sex was notably lower than in the Harlem or Bangkok arms, as those groups had median probability of 29 and 36% in the prescribed 2–24 hour window before sex and median probability of 31 and 55% in the 0–2 hours before sex without a previous pill, respectively [29].

Notably, we found no meaningful correlation between adherence to fixed and to on-demand pills (p = 0.84, Fig 1A). Number of sex acts was correlated with PrEP adherence only in the non-daily trial arms (p = 0.70, 0.02, 0.03 for daily, event-driven, and time-driven PrEP, respectively, Fig S5 in S1 File).

### Predicted effectiveness with single regimens

**Fig 2:**
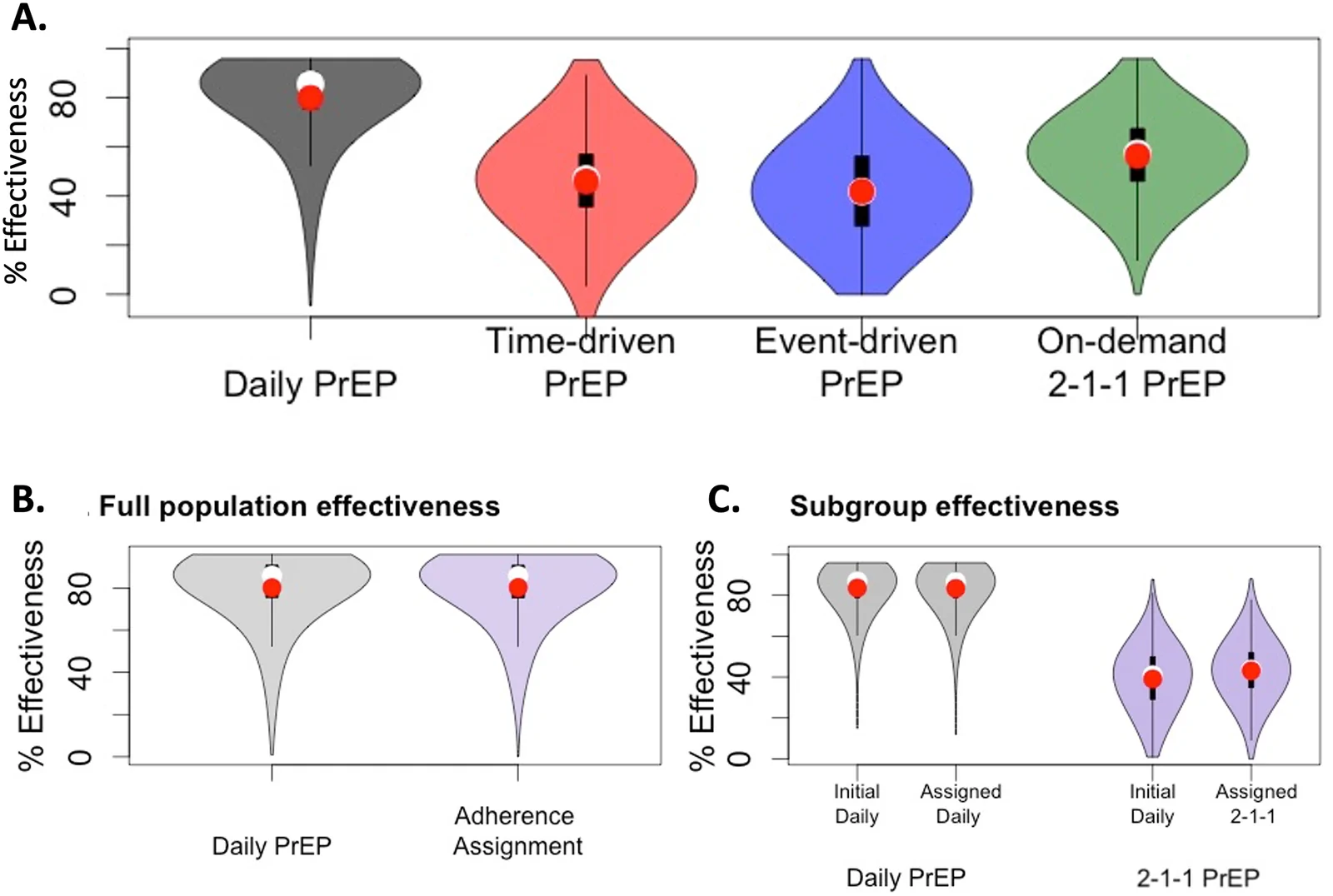
Estimated PrEP effectiveness. A: Overall PrEP effectiveness projected if all individuals are assigned to one of the HPTN 067 regimen or to 2-1-1 PrEP. B: Overall PrEP effectiveness projected if all individuals are assigned to daily PrEP (gray) compared to PrEP assignment personalized by adherence level (purple). C: Estimated PrEP effectiveness on different PrEP regimens among the group selected for daily PrEP (gray) and 2-1-1 PrEP (purple) use by personalized PrEP assignment with effectiveness in the initial daily assignment (left) and effectiveness when using their personalized PrEP assignment (right). *Colored bubbles show kernel density. Thick black bars show interquartile range. Red and white dots (may overlap) indicate mean and median values, respectively.*

Modeled effectiveness varied greatly when all individuals were assigned the same regimen (Fig 2A). Median effectiveness was highest for daily PrEP at 86% with median 5.6 pills taken per week. 2-1-1 PrEP had 57% median effectiveness with median 1.7 pills/week. Effectiveness was lowest for time- and event-driven PrEP tested in HPTN 067 with median effectiveness 47% and 42%, respectively, and median 2.3 and 1.7 pills/week, respectively.

### Predicted effectiveness with personalized regimens

Next, we compared scenarios between daily PrEP and a personalized adherence-based PrEP assignment where 2-1-1 PrEP was offered to the 8% of the population who (when assigned to daily PrEP) took daily PrEP pills less than 40% of the time (<2.8 pills/week). Our model projected 86% median population predicted effectiveness (Fig 2B). We projected modest effectiveness improvement for the small group of individuals assigned to 2-1-1 PrEP compared to when they used daily PrEP (median 44% vs. 41% effectiveness, Fig 2C). Notably, substantially fewer pills were taken by this group when using 2-1-1 PrEP (median 2.2 pills/week on daily PrEP vs. 1.4 pills/week on 2-1-1 PrEP).

### Sensitivity analyses

We conducted a sensitivity analysis using PrEP adherence-efficacy values from Marrazzo et al. [20] and 100% efficacy when 2-1-1 PrEP was taken correctly. Median 2-1-1 PrEP effectiveness rose slightly to 63% and median daily PrEP effectiveness was comparable at 86%. We further conducted a sensitivity analysis using minimum PrEP adherence-efficacy values from Moore et al. [19]. Here, median 2-1-1 PrEP effectiveness fell to 34% and median daily PrEP effectiveness fell to 54%.

We conducted a pharmacokinetic sensitivity analysis excluding 20 out of 178 participants who reported taking 2 + pills in the past week but with pharmacokinetic data showing zero drug detected in at least one test in that time. When these participants were removed from the analysis, the overall median effectiveness in the adherence-based assignment scenario was 86%. Six percent of individuals were assigned to 2-1-1 PrEP with personalized PrEP assignment and their median effectiveness rose from 40% on daily PrEP to 42% with 2-1-1 PrEP.

We also conducted a sensitivity analysis testing a higher alternate daily PrEP adherence threshold of 57% adherence (corresponding to taking 4 pills/week) in the adherence-based assignment scenario. If individuals taking fewer than 57% of daily PrEP pills were assigned 2-1-1 PrEP, it would be assigned to 20% of simulated individuals, 13 pp more than when the adherence threshold was 40%. Overall median population effectiveness under this assignment was comparable at 85% and the effectiveness for the subgroup assigned 2-1-1 PrEP was comparable to the original adherence-based assignment at 44%.

Finally, in our “optimal” PrEP assignment sensitivity analysis, we tried to balance estimated individual effectiveness and pill burden (see Supplement for details). Our model projected almost identical effectiveness for both personalized PrEP assignments, with 86% median population predicted effectiveness (Fig S6A in S1 File). There were modest effectiveness improvements in the 8% of individuals offered 2-1-1 PrEP than when using daily PrEP (median 48% vs. 43% effectiveness, Fig S6B in S1 File). Our optimal assignment analysis suggested that 2-1-1 PrEP provided benefits mainly to individuals with low expected adherence to daily PrEP while sex act frequency had little influence on projected regimen optimality (Fig S6C-D, Table S2 in S1 File).

## Discussion

We projected the effectiveness in reducing the risk of HIV acquisition for cisgender women in the daily and non-daily PrEP regimens tested in HPTN 067 with a simulation model. This trial provided a unique setup to study the pill-taking patterns of participants assigned to different oral PrEP options. HPTN 067 was not powered to capture differences in HIV incidence by study arm but reported PrEP coverage of sex acts, defined as at least one pill taken within four days before sex and one pill taken within one day after sex, as primary outcomes. Although an imperfect predictor of effectiveness, this metric suggested that participating women at the Cape Town study site achieved much better coverage on daily PrEP compared to time-driven or event-driven PrEP (75%, 56%, 52% of reported sex acts, respectively) [26]. The adherence reported in the study confirmed this order of regimen coverage level in the daily, time-driven, and event-driven PrEP arms (75%, 65%, 53% of prescribed pills taken, respectively). However, it was unclear how these metrics would translate into PrEP regimen effectiveness.

Our analysis, utilizing a recently published adherence-efficacy curve specific to women [19], addressed this question. We estimated that the effectiveness of daily oral PrEP at the Cape Town site of HPTN 067 was higher than either calculated sex-act coverage or reported adherence [26]. Our results also suggest that the effectiveness of time-driven and event-driven PrEP were projected to be substantially lower than the adherence and coverage metrics, resulting in a larger effectiveness gap between the daily and non-daily regimens. Notably, we were also able to predict the effectiveness of 2-1-1 PrEP, which was expected to outperform the other non-daily regimens by 10–15 percentage points (pp) when used by the female participants in HPTN 067, though with substantially lower effectiveness than daily PrEP.

Given the overwhelming advantage of daily PrEP among this population, we applied a person-centered approach aiming to identifying subgroups of cisgender women who may benefit from using 2-1-1 PrEP instead of daily PrEP. In our model, 2-1-1 PrEP was beneficial for the 8% of the cisgender women trial population in Cape Town who showed low adherence to daily PrEP and led to slightly increased overall effectiveness within this subgroup and required fewer pills per week, although population-level effectiveness remained unchanged. Early PrEP discontinuation is high, especially in Sub-Saharan Africa [32,33], which may make it difficult to change dosing schedules before an individual discontinues PrEP. However, one meta-analysis found that 37% of the people who discontinued PrEP later re-initiated it within one year of first initiation [32]. Discussion with a provider at re-initiation may be one place in which alternate dosing schedules could be introduced to those who have tried daily PrEP and could take place within existing PrEP programs.

In this trial population, assigning 2-1-1 PrEP by adherence to daily PrEP did not influence the overall population effectiveness compared to the scenario where all women were assigned to daily PrEP. Relatively few individuals were projected to benefit from 2-1-1 PrEP, mainly due to the uniformly high adherence reported for daily PrEP and lower adherence projected for on-demand PrEP at the HPTN 067 Cape Town site. In comparison, the same analysis examining the other HPTN 067 sites estimated that the optimality assignment may benefit up to a third of the MSM populations enrolled [29]. Notably, women had comparable daily PrEP adherence to MSM in Harlem and Bangkok. However, their on-demand adherence was significantly lower in both pre-sex intervals (in the 2−24 hours prior to sex and 0−2 hours prior to sex with no pill taken in the previous interval). For the women in Cape Town, this substantially reduced the likelihood of increasing individual-level effectiveness when assigned to 2-1-1 PrEP. Results may differ in other populations where women have demonstrated substantially lower adherence to daily oral PrEP [28]. For women who are adherent to the non-daily PrEP options, higher numbers of sex acts would also increase regimen effectiveness as pill taking becomes closer to that in the daily regimen.

While the pill taking and sex act behaviors we have modeled are specific to this population and may be difficult to determine outside of this trial, our finding that the individuals who may get the most benefit from 2-1-1 PrEP are those with low adherence to daily PrEP has been supported across the three distinct populations enrolled in the HPTN 067 study. One limitation of our model was that we were not able to use data directly from HPTN 067 in our modeling of 2-1-1 PrEP as it was not tested in the trial but instead used data from the event-driven regimen. This could lead to our adherence estimates being lower than if we had actual 2-1-1 data, as event-driven PrEP has a smaller post-sex window for adherence than 2-1-1- PrEP (2 hours vs 24 hours). A further limitation is in the generalizability of our behavioral data. The HPTN 067 trial occurred over a decade ago (enrollment occurred between September 2011 and October 2012) and adherence to daily oral PrEP was unusually high. The latter feature means that our estimation of the benefit of 2-1-1 PrEP is conservative and that there may be greater benefit to this regimen in other populations. While the behavioral data is older, this kind of detailed data on both pill-taking and sex acts is not commonly collected which adds to its value. Another limitation comes from assumptions in estimating PrEP efficacy from total pills taken before and after sex acts without regard for pill timing and in assigning uniform HIV risk per sex act across all individuals and their sex acts. This assumption gives equal effectiveness weight to the loading dose and subsequent two doses in 2-1-1 PrEP, even though macaque models suggest the loading dose may be more important for protection [34,35]. This may make our 2-1-1 effectiveness estimates overly optimistic. We used a generalized linear mixed model while other methods, such as a mixture distribution, may classify the distribution of pill taking more accurately as observed in Marrazzo et al.’s reanalysis of post-approval studies [20]. We also used a single efficacy value for each level of PrEP pills taken per week instead of incorporating more uncertainty into our main analysis. In our sensitivity analyses, modeling PrEP effectiveness at the upper bound of PrEP efficacy led to very little difference in our estimates. In contrast, modeling PrEP effectiveness at the lower bound of PrEP efficacy led to much lower estimates. Other modeling studies have estimated PrEP efficacy in cisgender women either consistently with or with higher efficacy than in our main analysis, [13] though all models incorporate wide confidence intervals. We also do not consider community viral load or HIV prevalence, as our model simulates which sex acts are protected, not risk of HIV acquisition. Women in Africa face multiple challenges when offered PrEP, including perceived efficacy of other prevention methods, pill sharing and gender-based violence, that affect their ability to take PrEP pills regularly [36,37]. Further analysis is needed to better understand the relationship between PrEP pill taking and the risk of HIV acquisition in future models.

## Conclusion

With the approval of novel HIV prevention products and regimens, including long-acting PrEP and vaginal rings, the focus must shift from drug discovery to providing access and choices to those who need them most. Individuals considering different PrEP options should receive up to date information about the potential benefits from each option including honest data-driven assessment of the expected reduction in HIV risk associated with preference, sexual behavior patterns, and likelihood of adherence. Mathematical modeling analyses like the one presented here may be of help to guide decision-makers in how to achieve better effective population coverage with planned HIV programs, which is often disconnected with the estimated actual intervention coverage [38]. Our modeling analysis suggests that 2-1-1 PrEP could be a viable option for a subset of cisgender women struggling with daily oral PrEP. This regimen could be especially valuable as it could be implemented within existing oral PrEP programs. We hope that future trials will be conducted to demonstrate the benefits of 2-1-1 PrEP for cisgender women in the real world and quantify the effectiveness for future modeling efforts. Similar modeling studies can be performed to identify cisgender women who could benefit from other PrEP methods which will promote the person-centered approach to HIV prevention.

## Supporting information

S1 File S1-6 Fig; S1-2 Table. Supporting figures and tables. Titles include Fig 1. Distribution of sex acts in the past 6 months for women in the HPTN 067 trial. 2: Distribution of PrEP adherence in A: HPTN 067 data and B: simulated data. 3: Bridging between PrEP regimens. 4: Diagram of PrEP regimens for a typical week. 5: Number of sex acts in the past 6 months and adherence to PrEP in individual arms and all PrEP arms together. 6: Estimated PrEP effectiveness. Table 1: Pill taking probability by interval type with simulation equation variables. 2: Predicted proportion for whom on-demand 2-1-1 PrEP was optimal in daily PrEP adherence and sex act frequency subgroups. Supplementary details for the mathematical model are also included.
(DOCX)
