# Supplementary material for: Estimated benefits of providing on-demand pre-exposure prophylaxis options for cisgender women in South Africa: A modeling study
Source: PLoS One. 2026 Aug 27;21(8):e0357094. doi: 10.1371/journal.pone.0357094 (PMC13521357; doi:10.1371/journal.pone.0357094)
Supplement: Supplementary file 1 [file pone.0357094.s001.docx]

**Supplement:**

ESTIMATED BENEFITS OF PROVIDING ON-DEMAND PREP OPTIONS FOR CISGENDER WOMEN IN CAPE TOWN: A MODELING STUDY

Sarah E. Stansfield, Mia Moore, Marie-Claude Boily, James P. Hughes, Deborah Donnell, Linda-Gail Bekker, Dobromir T. Dimitrov

**1. Supplementary Figures and Tables**

**Figure S1: Distribution of sex acts in the past 6 months for women in the HPTN 067 trial.** Numbers show number of participants in each group. Mean = 36, median = 27, max=178 sex acts.

**Figure S2: Distribution of PrEP adherence in A: HPTN 067 data and B: simulated data.** HPTN 067 data mean = 0.58, median =0.57. Simulated data mean = 0.58, median = 0.56.

*Figures S3&4 as referenced in the main manuscript can be found in Section 2 below.*

**Figure S5: Number of sex acts in the past 6 months and adherence to PrEP in individual arms and all PrEP arms together.** P-values shown are the product of linear regression.

*Figure S6 as referenced in the main manuscript can be found in Section 2 below.*

**Table S1: Pill taking probability by interval type with simulation equation variables.** A: Daily PrEP pill taking probabilities, estimated with HPTN 067 daily arm data. B: On-demand PrEP pill taking probabilities estimated with HPTN 067 event-driven arm data, except for the 2-24 hours prior to sex interval, which had probability assigned from 2-1-1 PrEP adherence data.

|  | Interval Type | Parameter | | Pill Taking Probability | |
| --- | --- | --- | --- | --- | --- |
|  |  | In Fig S2.1 | In equations | Median | IQR |
| A. Daily PrEP | Pill taken day before | D1 | $D_{1j}$ | 84 % | 71 - 92 % |
|  | Pill not taken day before | D2 | $D_{2j}$ | 63 % | 49 - 75 % |
| B. 2-1-1 PrEP | No sex act day before, day of, day after | O1 | $O_{1j}$ | 6 % | 3 - 11 % |
|  | 2-24 hours prior to sex act | O2 | $O_{2j}$ | 23 % | 16 - 31 % |
|  | 0-2 hours prior to sex act, pill taken in 2-24 hours prior to sex interval | O3 | $O_{3j}$ | 18 % | 13 - 25 % |
|  | 0-2 hours prior to sex act, pill not taken in 2-24 hours prior to sex interval | O4 | $O_{4j}$ | 19 % | 5 - 54 % |
|  | Day 1 after sex act, pre-sex act pill taken | O5 | $O_{5j}$ | 28 % | 15 - 48 % |
|  | Day 1 after sex act, pre-sex act pill not taken | O6 | $O_{6j}$ | 8 % | 3 - 18 % |
|  | Day 2 after sex act, day 1 after sex act pill taken | O7 | $O_{7j}$ | 90 % | NA |
|  | Day 2 after sex act, day 1 after sex act pill not taken | O8 | $O_{8j}$ | 8 % | 3 - 18 % |
| C. Time-driven PrEP | No pill for 2 days | T1 | $T_{1j}$ | 44% | 35-54% |
|  | Pill taken previous day or day before | T2 | $T_{2j}$ | 12% | 8-17% |
|  | Day after sex act with no 'after' pill | T3 | $T_{3j}$ | 26% | 20-32% |
|  | 0-2 hours before sex | T4 | $T_{4j}$ | 8% | 4-13% |
|  | 0-2 hours after sex | T5 | $T_{5j}$ | 26% | 16-38% |
| D. Event-driven PrEP | No sex act day before, day of, day after | E1 | $E_{1j}$ | 6 % | 3 - 11 % |
|  | 2-48 hours prior to sex act | E2 | $E_{2j}$ | 23 % | 16 - 31 % |
|  | 0-2 hours prior to sex act, pill taken in 2-48 hours prior to sex interval | E3 | $E_{3j}$ | 18 % | 13 - 25 % |
|  | 0-2 hours prior to sex act, pill not taken in 2-48 hours prior to sex interval | E4 | $E_{4j}$ | 19 % | 5 - 54 % |
|  | 0-2 hours after sex act, pre-sex act pill taken | E5 | $E_{5j}$ | 28 % | 15 - 48 % |
|  | Day after sex act, pre-sex act pill not taken | E6 | $E_{6j}$ | 8 % | 3 - 18 % |

**Table S2: Predicted proportion for whom on-demand 2-1-1 PrEP was optimal in daily PrEP adherence and sex act frequency subgroups.** Adherence cut-offs were approximately equal to quintiles while sex frequency cut-offs were approximately equal to tertiles*.*

| Adherence to Daily PrEP | % in Subgroup | % of Subgroup with  2-1-1 optimal |
| --- | --- | --- |
| 0-57% | 19.8% | 32.4% |
| 57-74% | 20.0% | 3.8% |
| 74-86% | 21.9% | 1.1% |
| 86-93% | 18.7% | 0.6% |
| 93-100% | 19.7% | 0.7% |
| Sex Acts past 6 months | % in Subgroup | % of Subgroup with  2-1-1 optimal |
| 0-15 | 33.2% | 8.4% |
| 15-32 | 33.5% | 6.5% |
| 32+ | 33.4% | 8.2% |

**2. Full Method Description**

***As our methods have been previously described [1], the methods description in sections 3 and 4 is reproduced verbatim here with updates only where necessary.***

Based on the observed patterns in HPTN 067, we developed an agent-based stochastic model simulating pill taking and sex act frequency and used the model to create a distribution of these behaviors for model agents assigned to daily and on-demand 2-1-1 PrEP. First, we estimated model parameters which govern pill taking behavior and sexual activity separately for each PrEP regimen in HPTN 067 (daily, time-driven, and event-driven). We then used these parameters to simulate on-demand PrEP by modifying the event-driven PrEP regimen with 2-1-1 PrEP guidelines^13^ (described further in section 2.1). PrEP adherence was measured separately for patterns of “fixed” pills taken daily or weekly at regular intervals and “on-demand” pills taken before and after sex acts observed in HPTN 067 (described further in section 2.2). Next, we created a synthetic trial population to simulate daily and on-demand PrEP and predict PrEP adherence for each modelled agent (described further in section 2.3). We calculated PrEP effectiveness for the synthetic trial population, measured as percent reduction in HIV risk, accounting for the predicted number of pills taken around agents’ sex acts (described further in section 2.4). We determined an optimal PrEP regimen for each agent based on calculated effectiveness and number of pills taken (described further in section 2.5). Finally, we evaluated the effectiveness of the optimal PrEP assignment and compared it to an adherence-based PrEP assignment in which only agents with low observed adherence to daily PrEP were assigned on-demand PrEP to determine the predicted impact of adding on-demand PrEP as an option (described further in section 2.6). Lastly, we validated adherence for HPTN 067 participants based on observed pharmacokinetic data (described further in 2.7).

Panel 1: model terms and definitions

| Model Terms | Definitions |
| --- | --- |
| Fixed regimen | PrEP pills taken at regular intervals (daily or weekly) |
| On-demand regimen | PrEP pills taken before and after sex acts |
| Optimal PrEP assignment | On-demand PrEP was assigned if it resulted in i) any improvement in effectiveness with fewer pills taken than daily PrEP (Criterion 1); ii) more than 10 percentage points (pp) improvement in effectiveness compared to daily PrEP (Criterion 2); or iii) less than 10 pp reduction in effectiveness compared to daily PrEP with fewer than half the number of pills taken (Criterion 3).  In all other cases daily PrEP was assigned. |
| Adherence-based PrEP assignment | Agents with adherence to daily PrEP below 40, 50, or 60% were assigned on-demand PrEP.  All other agents were assigned to daily PrEP. |

## 2.1 Creation of Synthetic Population

We created a synthetic population of 10,000 agents with defined pill taking and sex act frequency for each regimen $u_{R,j}$ ={$u_{Daily,j}$,$u_{Time,j}$,$u_{Event,j}$,$u_{211,j}$}.

To create each of these populations, we first sampled 10,000 adherence parameter sets $u_{R,j}$ from each regimen and site-specific parameter distribution. Adherence parameter sets were stochastically combined.

When an agent in the model was created, we sampled a participant from the time-driven arm of HPTN 067 (Figure S2.2). That participant had both fixed and on-demand PrEP adherence values in quintiles *x* and *y*, respectively. For fixed adherence, a participant in the daily PrEP arm with adherence in quintile *x* was sampled and the modeled agent had daily PrEP adherence equal to that participant. For on-demand adherence, a participant in the on-demand PrEP arm with post-sex pill adherence in quintile *y* was sampled and the modeled agent had on-demand PrEP adherence equal to that participant. In this way, we directly used observed adherence data from the daily and event-driven arms of HPTN 067, linked by time-driven arm participants’ propensities to adhere to fixed and on-demand pills. Note that model agents may or may not have fixed and on-demand adherence in the same quintile. Pill taking behavior at the given adherence level was modeled as described above.

**Figure S3: Bridging between PrEP regimens**. Plots show data from the HPTN 067 trial. A: Fixed and on-demand pill adherence in the time-driven arm of HPTN 067. Each point shows one participant’s adherence values. When a model agent is created, a participant from the time-driven arm of HPTN 067 is sampled (Participant X). B: Fixed and on-demand pill adherence in the time-driven arm sorted by increasing adherence. Participant X has fixed and on-demand PrEP adherence values in quintiles 3 and 4 respectively. C: Fixed pill adherence in the time-driven arm and fixed pill adherence in the daily PrEP arm, sorted by increasing adherence. For fixed adherence, a participant in the daily PrEP arm with adherence also in quintile 4 is sampled (Participant Y) and the modeled agent has daily PrEP adherence equal to that participant. D: On-demand pill adherence in the time-driven arm and on-demand pill adherence in the event-driven arm, sorted by increasing adherence. For on-demand adherence, a participant in the event-driven PrEP arm with post-sex pill adherence in quintile 3 is sampled (Participant Z) and the modeled agent has on-demand PrEP adherence equal to that participant.

## 2.2 Modeling pill-taking and sex act frequency

Using observed data from HPTN 067 [2], we estimated individual pill-taking by dividing the study follow-up time into discrete adherence intervals during which a pill may or may not be taken. These intervals ranged from 2-44 hours and depended on proximity to sex acts. Intervals were grouped into interval types, depending on the assigned PrEP regimen (daily or on-demand), recent pill-taking behavior, and proximity to sex acts (Figure S2.1). $P_{ij}$ refers to the probability of individual $j$ taking PrEP during interval $i$. This depended on $u_{R,j},$ the PrEP adherence parameter for individual $j$ and regimen $R;$ $u_{R,j}$ had one entry per interval type and referred to the log-odds of taking a pill (see Figure S2.1 for interval definitions). Its distribution was estimated from HPTN 067 data.

$$\begin{matrix} \begin{matrix} \text{Individual parameters: Regimen R, individual j} \text{u}_{R,j}\in\mathbb{R}^{N_{T}} & \sim N(\beta_{R},\Sigma_{R}) \\ \text{Pill taking probability in interval i} \text{logit}(P_{i,j}) & =u_{R,j,Type_{i}} \end{matrix} \end{matrix}$$

The mean, $\beta_{R}$, and covariance matrix, $\Sigma_{R}$, describe the population distribution of the PrEP adherence parameters (see Table 1 in main manuscript) for regimen $R$ and can be used to simulate a synthetic population. The number of modeled sex acts for each agent on each day was Poisson distributed with agent-specific mean $\mu_{j}$. This setup allows for an overdispersed, heavy tailed distribution which accounts for differences in sexual activity between individuals. $\beta_{R}$, $\Sigma_{R}$, and $\mu_{j}$ were estimated from HPTN 067 data [2].

**Figure S4: Diagram of PrEP regimens for a typical week**. A: daily PrEP: one pill per day; on-demand PrEP: two pills 2-24 hours before sex acts (only one pill if other pills taken in previous seven days), one pill 24 hours after the last pre-sex pill, and one pill 24 hours after that. B: Diagram of PrEP regimens’ adherence interval types. Each regimen’s adherence interval depended on previous pills taken (outlined label boxes) and on time before or after sex acts. Pill taking and sex acts here are to illustrate when each adherence interval occurs and do not reflect actual data.

### 2.2.1 Daily PrEP

For daily PrEP, there were two interval types, D1 and D2, corresponding to days with or without a pill taken on the previous day. Pill taking probabilities were calculated from the observed daily arm of HPTN 067.

### 2.2.2 On-demand PrEP

We extended the probability estimates based on data from observed event-driven PrEP to inform adherence probabilities for on-demand 2-1-1 PrEP assuming individuals were as likely to adhere to the on-demand 2-1-1 regimen as they were to the event-driven regimen in HPTN 067. Therefore, we used pre-sex, post-sex, and make-up (an otherwise unneeded pill taken the day after a prescribed pill was missed) pill taking probabilities from the observed event-driven arm of HPTN 067 (see supplement for full details) to simulate on-demand 2-1-1 PrEP. The modeled pre-sex pill was doubled to two pills if there were no other pills taken in the previous seven days or current day, reflecting that 2-1-1 users are instructed not to double their first pill if they have taken pills in the preceding week. If sex acts occurred on multiple consecutive days, only one modeled pill would be taken on each subsequent day. The modeled make-up pill was doubled under the same conditions as the pre-sex pill, reflecting instructions to take two pills after sex if the pre-sex pills were missed [3]. If the first post-sex pill was taken, the second modeled pill was taken on the following day with a 90% probability [4]. An alternative scenario in which the second post-sex pill was independent of the first (with the same pill taking probability as the first post-sex pill) was also simulated. If the first post-sex pill was missed, the second post-sex pill was taken with the make-up pill probability.

### 2.3 Estimating PrEP Adherence

Two measures of adherence were used for mapping between regimens: “fixed” pill adherence covering daily or weekly pills which were expected to be taken at regular intervals and “on-demand” pill adherence which were expected to be taken before and after sex acts. We evaluated the Pearson correlation coefficient between adherence to fixed and on-demand pills using data from the time-driven arm of HPTN 067 where participants took both fixed and on-demand pills so adherence to both types could be assessed in the same participants in the same time-period. We assumed agents retained the same fixed pill adherence quintile when assigned to time-driven PrEP or daily PrEP and the same on-demand pill adherence quintile when assigned to time-driven PrEP or on-demand PrEP. This allowed us to predict agent adherence levels when assigned to different regimens.

**2.4	Estimating PrEP Effectiveness**

PrEP effectiveness was calculated on a per-sex act basis for each model agent. For sex act k, we added together the number of pills taken on the day of, the four days prior, and the two subsequent days. If at least two, four, or seven cumulative pills were taken, we assumed that the risk of HIV acquisition for that sex act was reduced by $\varepsilon_{k}=$59.3%, 83.8%, or 95.9%, respectively [5].

Each sex act had an intrinsic untreated hazard, $\rho_{k},$ depending on condom use, sexual positioning, STI status of the agent, etc. This hazard for sex act k was reduced to $\rho_{k}\left( 1 -\varepsilon_{k} \right)$ during treatment. We defined the predicted effectiveness value for an agent to be the reduction in cumulative HIV hazard compared to no PrEP

$$Effectiveness for an agent = 1 - \frac{\sum_{k} \rho_{k}\left( 1 -\varepsilon_{k} \right)}{\sum_{k} \rho_{k}}$$

We assumed the untreated intrinsic hazard $\rho_{k}$ was constant across all of an individual’s sex acts or, equivalently, that their protection $\varepsilon_{k}$ was uncorrelated with $\rho_{k}.$ With this assumption, the effectiveness for an agent simplified to the mean level of protection across all sex acts. The population-level effectiveness was similarly the mean level of protection across all sex acts in the synthetic population.

## 2.5 Adherence-based PrEP assignment

We evaluated a simplified, adherence-based PrEP assignment constructed on the simulated adherence to daily PrEP only. In this analysis, we simulated the entire synthetic population on daily PrEP for six months and calculated each agent’s adherence (as the mean number of days a pill was taken). On-demand PrEP was assigned to agents with daily PrEP adherence below 40% while daily PrEP was assigned to all other agents. The synthetic population was simulated for another six months. A sensitivity analysis were conducted with on-demand PrEP assigned to agents with daily PrEP adherence <57%.

**2.6 Pharmacokinetic Validation of Adherence (Sensitivity Analysis)**

To determine whether observed real-time electronic drug monitoring pill bottles and self-reported pill taking in HPTN 067 was accurate, we examined pharmacokinetic data consisting of peripheral blood mononuclear cell (PBMC) concentrations of tenofovir diphosphate (TVF-DP) to determine whether there were mismatches between reported pill taking and measured drug concentrations. Mismatched adherence was defined as a report in which two or more pills were reportedly taken in the past week, but drug concentration was measured as zero.

## 2.7 Optimal PrEP Assignment (Sensitivity Analysis)

We modeled a second personalized PrEP regimen. We evaluated the effectiveness of an “optimal” PrEP assignment in which we assign either daily or 2-1-1 PrEP for each agent based on calculated individual effectiveness and estimated difference in pill burden. Regimen optimality was determined by a mix of effectiveness and pill burden criteria with 2-1-1 PrEP chosen if it provided: i) any improvement in effectiveness with fewer pills taken than daily PrEP (Criterion 1); ii) >10 percentage points (pp) higher effectiveness than daily PrEP (Criterion 2); or iii) <10 pp lower effectiveness than daily PrEP with fewer than half the number of pills taken (Criterion 3). In all other cases daily PrEP was identified as optimal. These criteria were chosen to reflect the potential preference of individuals to take fewer pills as long as effectiveness was not greatly reduced. We evaluated an optimal PrEP assignment in which each of the 10,000 agents in the synthetic population was assigned to their optimal regimen and simulated for six months.

In the optimal assignment scenario, 2-1-1 PrEP was offered to the 8% of the population who were projected to do better on it (based on the optimal assignment criteria above). Our model projected almost identical effectiveness for universal daily PrEP and both personalized PrEP assignments (adherence-based and optimal), with 86% median population predicted effectiveness (Figure 2.3A).

In the optimal assignment there was also modest effectiveness improvements in the subgroup of agents receiving 2-1-1 PrEP than when using daily PrEP (median 48% vs. 43% effectiveness, Figure 2.3B). The projected gap in expected pill burden for this population was similarly large (median 2.4 pills/week on daily PrEP vs. 1.5 pills/week on 2-1-1 PrEP). At the individual level, effectiveness was expected to increase for 77% of the group while 75% were expected to take fewer pills with 2-1-1 PrEP.

Our optimal assignment analysis suggested that 2-1-1 PrEP provided benefits mainly to individuals with low expected adherence to daily PrEP. Sixty-two percent of those who would take less than 40% of their daily pills were assigned to 2-1-1 PrEP (Figure S2.3C, Table S1.2), while daily PrEP was optimal for 99% of the agents who would take more than 75% of their daily pills when assigned to daily PrEP. Benefits from assignment to 2-1-1 PrEP varied by estimated daily PrEP adherence. Ninety-two percent of agents with low adherence to daily PrEP (below 40%) for whom 2-1-1 PrEP was optimal were assigned to it because it improved their effectiveness. All agents with good daily PrEP adherence (above 75%) for whom 2-1-1 PrEP was optimal were assigned to it based on pill reduction. We found that sex act frequency had little influence on projected regimen optimality (Figure S2.3D, Table S1.2). We expected that agents with more frequent sex acts will be less likely to benefit from using 2-1-1 PrEP, but differences across sexual activity strata were insignificant.

**Figure S6: Estimated PrEP effectiveness.** A: Overall PrEP effectiveness projected if all agents are assigned to daily PrEP compared to personalized PrEP assignment. B: Estimated PrEP effectiveness on different PrEP regimens among the group selected for 2-1-1 PrEP use by personalized PrEP assignment. *Colored bubbles show kernel density. Thick black bars show interquartile range. Red and white dots (may overlap) indicate mean and median values, respectively.* **C & D. Predicted numbers of agents in each quintile of adherence to daily PrEP (C) and tertile of sex frequency (D)**. **Colored portions of bars show agents for whom 2-1-1 PrEP was optimal.** Optimality categories: Overall Better (purple): any improvement in effectiveness with fewer pills taken than daily PrEP; Higher Efficacy (blue): >10 percentage points (pp) higher effectiveness than daily PrEP; Fewer Pills (red): <10 pp lower effectiveness than daily PrEP with fewer than half the number of pills taken.

**3. Simulation Equations**

An individual’s risk of HIV infection depends on their frequency of sexual activity ($\mu_{j}$) and their individualized PrEP adherence parameters $\text{u}_{R,j}$. Both sexual and pill taking parameters are drawn from empirical distributions (see main text and Table S1.1).

To evaluate risk on a regimen, individuals’ sexual activity and pill-taking is simulated each day $i=1\ldots180$. The number of sex acts on day $i$ is poisson distributed with mean $\mu_{j}$.

$$\begin{matrix} S_{ij}\sim\text{Pois}(\mu_{j}) \end{matrix}$$

To estimate effectiveness, we calculate the number of pills taken each day, $P_{ij}$, the number of sex acts with a post-sex pill taken within two hours $S_{ij}^{\text{post}}$, and whether or not acts on day $i$ are covered by a “pre-pill” taken 2-48 hours ahead of time, $N_{ij}$. The calculation of these three quantities is different for each regimen (see subsequent section).

For all regimens, PrEP effectiveness primarily relies on the rolling weekly sum of the number of pills taken.

$$\begin{matrix} \text{Rolling Weekly Pills}\text{ }W_{ij} & =\sum_{k=i-4}^{i+2} P_{ij} \end{matrix}$$

However, if fewer than four pills are taken, but they include a properly taken pre- and post-sex pill, the PrEP effectiveness is increased. Specifically, the per-act effectiveness of PrEP is calculated using the following formula.

$$\begin{matrix} \text{Single act efficacy}\phi(W,N,\text{Po}) & =\left\{ \begin{matrix} 0 & W<2 \\ 0.593 & 2\leq W<4\text{ and }(N=1\text{ or Po}==0) \\ 0.838 & 4\leq W<7\text{ or }\left( W<7\text{ and }(N=0\text{ and Po}==1) \right) \\ 0.959 & W\geq7 \end{matrix} \right. \end{matrix}$$

Finally, PrEP effectiveness is averaged across all sex acts.

$$\begin{matrix} \text{PrEP effectiveness} & =\frac{\sum_{i} \phi(W_{ij},N_{ij},0)(S_{ij}-S_{ij}^{\text{post}})+\phi(W_{ij},N_{ij},1)(S_{ij}^{\text{post}})}{\sum_{i} S_{ij}} \end{matrix}$$

## 3.1 Daily PrEP

In the daily PrEP regimen, the regimen specific parameters contains two parameters, $\text{u}_{D,j}=[D_{1j},D_{2j}]$, corresponding to the probability of taking a pill if one was or was not taken the day before, respectively. We assume that only one pill can be taken on any given day and that it has a 1/12 chance of falling with the two-hour window to provide post-sex protection. Adherence in the daily arm is defined as the number of days with at least one pill taken.

$$\begin{matrix} \rho_{ij} & =\left\{ \begin{matrix} D_{1j} & P_{i-1,j}=1 \\ D_{2j} & P_{i-1,j}=0 \end{matrix} \right. \\ P_{ij} & \sim\text{Binom}(1,\rho_{ij}) \\ S_{ij}^{\text{post}} & =\text{Binom}(1,\rho_{ij}(1-(11/12)^{(}S_{ij}))) \\ N_{ij} & =(P_{ij}==0)\text{and}(P_{i-1,j}==0) \\ \text{Adherence.Daily} & =\frac{1}{180}\sum I(P_{ij}\geq1) \end{matrix}$$

## 3.2 Time-driven PrEP

In the time-driven PrEP regimen, the regimen specific parameters contains five parameters, $\text{u}_{T,j}=[T_{1j},T_{2j},\ldots,T_{5j}]$.

First, we simulate pill-taking immediately before and after sex. Even though the time-driven PrEP regimen only called for pills directly following sex, individuals were also more likely to take pills immediately before sex acts regardless of how many pills had already been taken. Therefore, we categorize all sex acts by whether a post-sex pill was taken, a pre-sex pill was taken, or both. Note that we limit the total number of “post-pills” and “pre-pills” on a given day to one each as participants were instructed to avoid taking excessively many pills in a given day

$$\begin{matrix} S_{ij}^{\text{post}} & =\text{min}(1,\text{Binom}(S_{ij},T_{5j})) \\ S_{ij}^{\text{both}} & =\text{Binom}(S_{i}^{\text{post}},T_{4j}) \\ S_{ij}^{\text{post only}} & =S_{ij}^{\text{post}}-S_{ij}^{\text{both}} \\ S_{ij}^{\text{pre only}} & =\text{min}(1,\text{Binom}(S_{ij}-S_{ij}^{\text{post}}),T_{4j}) \end{matrix}$$

The time-driven regimen also required individuals to regularly take pills not associated with any sex acts. In practice, we found that individuals were more likely to take pills when it had been at least two days since they had taken any or if on the previous day they had missed a required post-sex pill. Using this, we iteratively simulate the number of pills taken on

$$\begin{matrix} \rho_{i} & =\left\{ \begin{matrix} T_{1j} & P_{i-1,j}+P_{i-2,j}=0 \\ T_{2j} & \left( P_{i-1,j}+P_{i-2,j}>0 \right)\text{and}\left( S_{ij}=S_{ij}^{\text{post}} \right) \\ T_{3j} & \left( P_{i-1,j}+P_{i-2,j}>0 \right)\text{and}\left( S_{ij}>S_{ij}^{\text{post}} \right) \end{matrix} \right. \\ P_{ij} & =\text{Binom}(1,\rho_{i})+S_{ij}^{\text{pre only}}+S_{ij}^{\text{post only}}+2*S_{ij}^{\text{both}} \\ N_{ij} & =\left( P_{ij}=0\text{ and }P_{i-1,j}=0 \right) \end{matrix}$$

Adherence in the time-driven arm, is defined separately for the regular pills and on-demand pills. Regular pill adherence is defined as the fraction of week with at least two pills taken.

$$\begin{matrix} \text{Fixed adherence} & =\frac{1}{25}\sum_{w=1}^{25} I\left( W_{7i,j}\geq2 \right) \end{matrix}$$

Post-pill adherence is defined as the proportion of sex acts with a post-pill, which is $T_{5j}$ by definition.

$$\begin{matrix} \text{On-demand adherence} & =T_{5j} \end{matrix}$$

## 3.3 Event-driven PrEP

In the event-driven PrEP regimen, the regimen specific parameters contains six parameters, $\text{u}_{E,j}=[E_{1j},E_{2j},\ldots,E_{6j}]$.

We first simulate the number of sex acts with and without post-sex pills on each day, with a maximum of one post-sex pill per day.

$$\begin{matrix} S_{ij}^{\text{post}} & =min(1,\text{Binom}(S_{ij},E_{6j})) \end{matrix}$$

We then simulate the pre-sex pills iteratively. These represent pre-sex pills taken 2-48 hours before sex. They can also include “make-up” pills, in which a pill is taken on the day following a missed post-sex dose.

$$\begin{matrix} \rho_{ij} & =\left\{ \begin{matrix} E_{1j} & (S_{ij}+S_{i+1,j}=0)\text{and}\left( S_{ij}=S_{ij}^{\text{post}} \right) \\ E_{2j} & \left( S_{ij}+S_{i+1,j}>0 \right) \\ E_{5j} & \left( S_{ij}+S_{i+1,j}=0 \right)\text{and}\left( S_{ij}>S_{ij}^{\text{post}} \right) \end{matrix} \right. \\ \tilde{P}_{ij} & =\text{Binom}(1,\rho_{ij})+S_{ij}^{\text{post}} \\ N_{ij} & =\left( \tilde{P}_{ij}=0\text{ and }\tilde{P}_{i-1,j}=0 \right) \end{matrix}$$

Finally, we simulate the pills taken 0-2 hours prior to sex. The rate at which these pills are taken depends on whether a pill was taken 2-48 hours prior. As with the post-sex pills, only one pre-sex pill can be taken on any given day.

$$\begin{matrix} S_{ij}^{\text{pre}} & =\left\{ \begin{matrix} min(1,\text{Binom}(S_{ij}-S_{ij}^{\text{post}},E_{3j})) & N_{ij}=0 \\ min(1,\text{Binom}(S_{ij}-S_{ij}^{\text{post}},E_{4j})) & N_{ij}=1 \end{matrix} \right. \\ P_{ij} & =\tilde{P}_{ij}+S_{ij}^{\text{pre}} \end{matrix}$$

Adherence to pre-sex pills is defined to be the proportion of sex acts that had a pill taken 0-48 hours beforehand. This includes pills which are taken “correctly”, 2-48 hours before, and those taken “late”, 0-2 hours. We assume that individuals taking pills “late” are adherent to the regimen, but have difficulty anticipating the need to take a pill ahead of time.

Adherence to post-sex pills is the probability of taking a pill in the 0-2 hours following sex, i.e. $E_{6j}$.

$$\begin{matrix} \text{Pre-sex adherence} & =\frac{\sum_{ij}^{180} I(N_{ij}==0)S_{ij}+I(N_{ij}==1)S_{ij}^{\text{pre}}}{\sum_{ij}^{180} S_{ij}} \\ \text{Post-sex adherence} & =E_{6j} \end{matrix}$$

## 3.4 On-demand 2-1-1- PrEP

Simulation of on-demand 2-1-1 PrEP is quite similar to time-driven PrEP, with a few minor additions. The regimen specific parameters are an extension of the event-driven parameters $\text{u}_{O,j}=[O_{1j},O_{2j},\ldots,O_{8j}]=[E_{1j},E_{2j},\ldots,E_{6j},O_{7j},O_{8j}]$. Calculation of the pre-sex and first post-sex pills is the same as in the event-driven regimen except that the pre-sex pill is doubled.

$$\begin{matrix} \tilde{P}_{ij} & =2\text{Binom}(1,\rho_{ij})+S_{ij}^{\text{post}} \end{matrix}$$

There is also second post-sex pill. If the first post-sex pill is taken, this second is taken with probability $O_{7j}$. If it is missed, the pill is treated as a “makeup” pill with probability $O_{8j}$

$$\begin{matrix} \hat{P}_{ij} & =\tilde{P}_{ij}+S_{ij}^{\text{pre}} \\ S_{ij}^{\text{post2}} & =(\hat{P}_{ij}==0)*min(1,\text{Binom}(S_{i-1,j}^{\text{post}},O_{8j}))+min(1,\text{Binom}(S_{ij}-S_{i-1,j}^{\text{post}},O_{8j})) \\ P_{ij} & =\hat{P}_{ij}+S_{ij}^{\text{post2}} \end{matrix}$$

**References**

1.	Stansfield, S.E., et al., *Estimating benefits of using on-demand oral prep by MSM: a comparative modeling study of the US and Thailand.* eClinicalMedicine, 2023. **56**: p. 101776.

2.	Bekker, L.G., et al., *Daily and non-daily pre-exposure prophylaxis in African women (HPTN 067/ADAPT Cape Town Trial): a randomised, open-label, phase 2 trial.* Lancet HIV, 2018. **5**(2): p. e68-e78.

3.	Foundation SFA. *Q&A: PrEP 2-1-1*. 2019; Available from: <https://www.sfaf.org/resource-library/qa-prep-2-1-1-for-anal-sex/>.

4.	Camp, C. and P. Saberi, *Facilitators and barriers of 2-1-1 HIV pre-exposure prophylaxis.* PLoS One, 2021. **16**(5): p. e0251917.

5.	Moore, M., et al., *Efficacy estimates of oral pre-exposure prophylaxis for HIV prevention in cisgender women with partial adherence.* Nat Med, 2023. **29**(11): p. 2748-2752.
